# Quantification of valvular regurgitation by transthoracic 3D high pulse repetition frequency Doppler echocardiography

**DOI:** 10.1093/ehjimp/qyae138

**Published:** 2024-12-18

**Authors:** Erik Andreas Rye Berg, Stefano Fiorentini, Jørgen Avdal, Bjørnar Grenne, Knut Haakon Stensæth, Peter Thomas While, Torvald Espeland, Rune Wiseth, Hans Torp, Svend Aakhus

**Affiliations:** Department of Circulation and Medical Imaging, Faculty of Medicine and Health Science, Norwegian University of Science and Technology (NTNU), Prinsesse Kristinas gate 3, Trondheim 7030, Norway; Clinic of Cardiology, St. Olavs Hospital, Trondheim University Hospital, Prinsesse Kristinas gate 3, Trondheim 7030, Norway; Department of Circulation and Medical Imaging, Faculty of Medicine and Health Science, Norwegian University of Science and Technology (NTNU), Prinsesse Kristinas gate 3, Trondheim 7030, Norway; Department of Circulation and Medical Imaging, Faculty of Medicine and Health Science, Norwegian University of Science and Technology (NTNU), Prinsesse Kristinas gate 3, Trondheim 7030, Norway; Department of Circulation and Medical Imaging, Faculty of Medicine and Health Science, Norwegian University of Science and Technology (NTNU), Prinsesse Kristinas gate 3, Trondheim 7030, Norway; Clinic of Cardiology, St. Olavs Hospital, Trondheim University Hospital, Prinsesse Kristinas gate 3, Trondheim 7030, Norway; Department of Radiology and Nuclear Medicine, St. Olavs Hospital, Trondheim University Hospital, Prinsesse Kristinas gate 3, Trondheim 7030, Norway; Department of Circulation and Medical Imaging, Faculty of Medicine and Health Science, Norwegian University of Science and Technology (NTNU), Prinsesse Kristinas gate 3, Trondheim 7030, Norway; Department of Radiology and Nuclear Medicine, St. Olavs Hospital, Trondheim University Hospital, Prinsesse Kristinas gate 3, Trondheim 7030, Norway; Department of Circulation and Medical Imaging, Faculty of Medicine and Health Science, Norwegian University of Science and Technology (NTNU), Prinsesse Kristinas gate 3, Trondheim 7030, Norway; Clinic of Cardiology, St. Olavs Hospital, Trondheim University Hospital, Prinsesse Kristinas gate 3, Trondheim 7030, Norway; Department of Circulation and Medical Imaging, Faculty of Medicine and Health Science, Norwegian University of Science and Technology (NTNU), Prinsesse Kristinas gate 3, Trondheim 7030, Norway; Clinic of Cardiology, St. Olavs Hospital, Trondheim University Hospital, Prinsesse Kristinas gate 3, Trondheim 7030, Norway; Department of Circulation and Medical Imaging, Faculty of Medicine and Health Science, Norwegian University of Science and Technology (NTNU), Prinsesse Kristinas gate 3, Trondheim 7030, Norway; Department of Circulation and Medical Imaging, Faculty of Medicine and Health Science, Norwegian University of Science and Technology (NTNU), Prinsesse Kristinas gate 3, Trondheim 7030, Norway; Clinic of Cardiology, St. Olavs Hospital, Trondheim University Hospital, Prinsesse Kristinas gate 3, Trondheim 7030, Norway

**Keywords:** valvular heart disease, valvular regurgitation, 3D Doppler, high pulse repetition frequency, transthoracic echocardiography

## Abstract

**Aims:**

To improve quantification of valvular regurgitation, a 3D high-pulse repetition frequency Doppler (3D HPRFD) method was developed for regurgitant volume (RVol) estimation from transthoracic echocardiography (TTE). Although successfully applied *in vitro* and in selected clinical cases, a systematic clinical validation of 3D HPRFD has not been published. Hence, our aims were to investigate (i) feasibility of 3D HPRFD and (ii) correlation between 3D HPRFD and RVol estimates obtained by the 2D proximal isovelocity surface area (PISA) method and cardiac magnetic resonance (CMR) in patients with either aortic regurgitation (AR) or mitral regurgitation (MR).

**Methods and results:**

We included 45 patients with AR (42% mild, 40% moderate, and 18% severe) and 45 with MR (67% mild, 24% moderate, and 9% severe). Median time between start of TTE and start of CMR was 1.5 h, minimizing changes in load. Overall feasibility of 3D HPRFD was 56% in AR and 44% in MR. Feasibility was only 25% in patients with severe regurgitation. In AR, estimated RVol from 3D HPRF did not correlate with estimated RVol from PISA or CMR [Spearman *rho* = 0.06 (*P* = 0.78) and 0.04 (*P* = 0.4), respectively]. In MR, RVol estimates from 3D HPRFD correlated with PISA (*rho* = 0.72, *P* < 0.001) but not with CMR (*rho* = 0.31, *P* = 0.43).

**Conclusion:**

Regurgitant volume estimation by 3D HPRFD had a low feasibility, especially in severe regurgitation, and in general correlated poorly with PISA and CMR estimates. In its current state, 3D HPRFD is not ready for clinical use.

## Graphical Abstract

**Graphical abstract:**
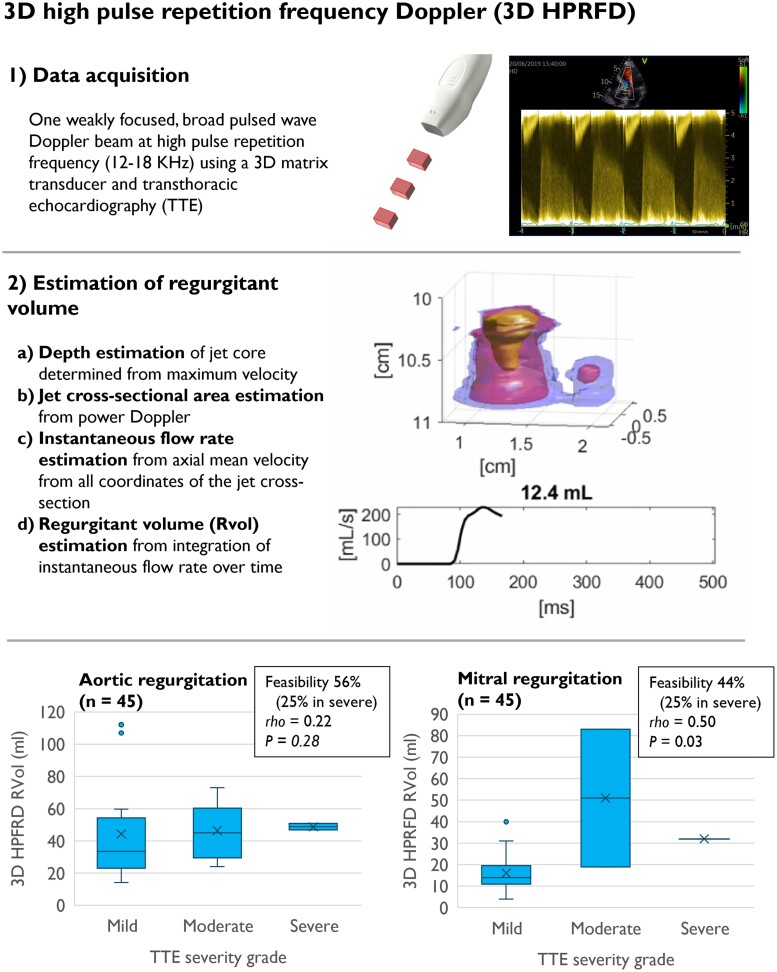

## Introduction

Echocardiographic assessment of valvular regurgitation requires comprehensive imaging and an integrative, multi-parametric approach.^1,2^ However, the modest reproducibility of essential parameters, including the proximal isovelocity surface area (PISA), is a concern.^3,4^ Furthermore, violation of PISA assumptions may lead to erroneous flow quantification.^5–7^ Finally, the multiple parameters included in the recommended comprehensive integrative approach are frequently discordant.^8^ This underlines the challenges of this approach and further questions reproducibility.

To address these challenges, advances in flow convergence-based quantification of regurgitant volume (RVol) have included temporal averaging of PISA to account for non-constant regurgitant flow rate,^9^ 3D PISA to account for a non-circular regurgitant orifice,^10^ and advanced non-linear 3D estimation of the flow convergence,^11^ the latter currently requiring transoesophageal echocardiography (TOE).

In parallel, regurgitation quantification from transthoracic echocardiography (TTE) based on the high-velocity jet core has been demonstrated through single and multibeam acquisitions.^12–15^ Although the results have been promising, and a potential for automation has been demonstrated,^16^ these methods are technically demanding, requiring power Doppler calibration in each recording.

Aiming to improve quantification of valvular regurgitation, a method for high-velocity flow quantification based on 3D high pulse repetition frequency Doppler (3D HPRFD) TTE has been developed. *In vitro*, flow estimation by 3D HPRFD correlated strongly with reference measurements.^17^ The method has been successfully applied in selected clinical cases. However, a systematic clinical validation of 3D HPRFD has not yet been published. Hence, the aims of our study were the following:

1. To investigate and report the feasibility of 3D HPRFD for RVol estimation in patients with aortic regurgitation (AR) and mitral regurgitation (MR).
2. To investigate and report correlation and agreement between RVol estimates from 3D HPRFD and 2D PISA RVol, cardiac magnetic resonance (CMR) RVol and overall TTE regurgitation severity grade.

## Methods

### Study population

We recruited AR and MR patients above 18 years from the outpatient clinic or scheduled for pre-surgical assessment at the Clinic of Cardiology, St. Olavs hospital, Trondheim, Norway. The exclusion criteria were contraindications to CMR; arrhythmia; BMI > 35 kg/m^2^; inability to lie flat; any metal close to the heart, including prosthetic valves, implantable cardiac devices, sternal cerclage and orthopaedic implants; or inability to undergo the scheduled exams.

### Data acquisition

Transthoracic echocardiography 3D HPFRD data were acquired using a Vivid e95 scanner with research-specific software and a 4V-D probe [GE Vingmed Ultrasound (GEVU), Horten, Norway]. Reference TTE data were acquired using a commercial Vivid e95 scanner and either a combination of M5Sc and 4V-D probes or a 4Vc-D probe (GEVU). Cardiac magnetic resonance data were acquired using a Prisma 3-Tesla scanner (Siemens Healthineers, Erlangen, Germany).

### 3D HPRFD

**Figure 1:**
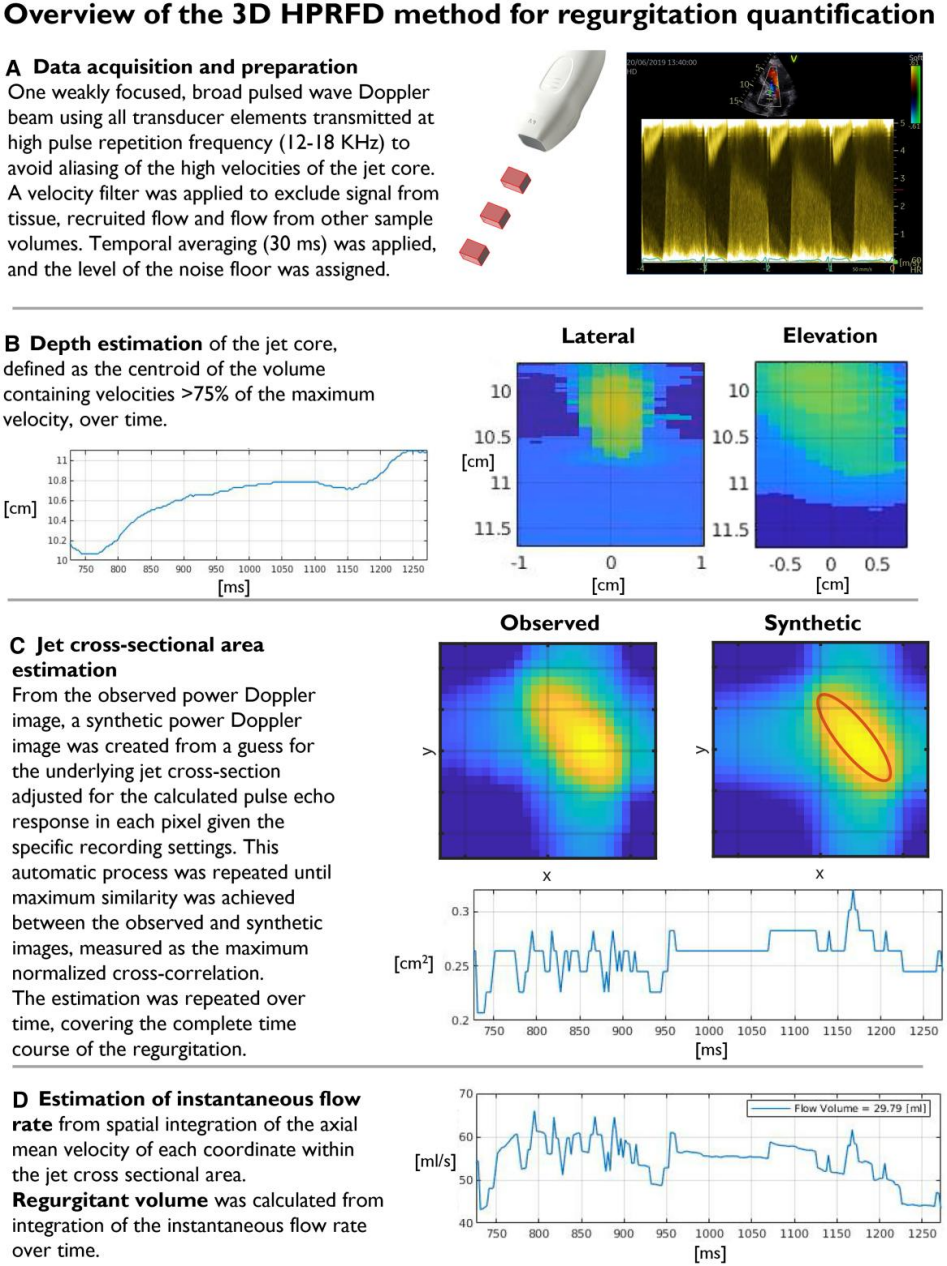
Overview of the 3D HPRFD method. *A*) A screenshot of the pulsed wave Doppler signal used for acquisition guidance. *B*) Maximum velocity maps from AR at early diastole are displayed to the right, where the brightest yellow colour corresponds to the highest velocities. The plot to the left displays estimated jet core depth over time. *C*) Examples of observed and synthetic cross-sectional power Doppler images of the jet core are displayed to the right. The red ellipsis displays the estimated jet cross-section. The plot displays estimated cross-sectional area over time. *D*) The plot displays instantaneous flow rate over time.

The method has previously been presented in detail.^17^ An overview is presented in *Figure 1*. In principle, the method calculates jet cross-sectional area for each frame, instantaneous flow rate through spatial integration of the velocities in the jet cross-sectional area, and RVol from temporal integration of the flow rate.

**Figure 2:**
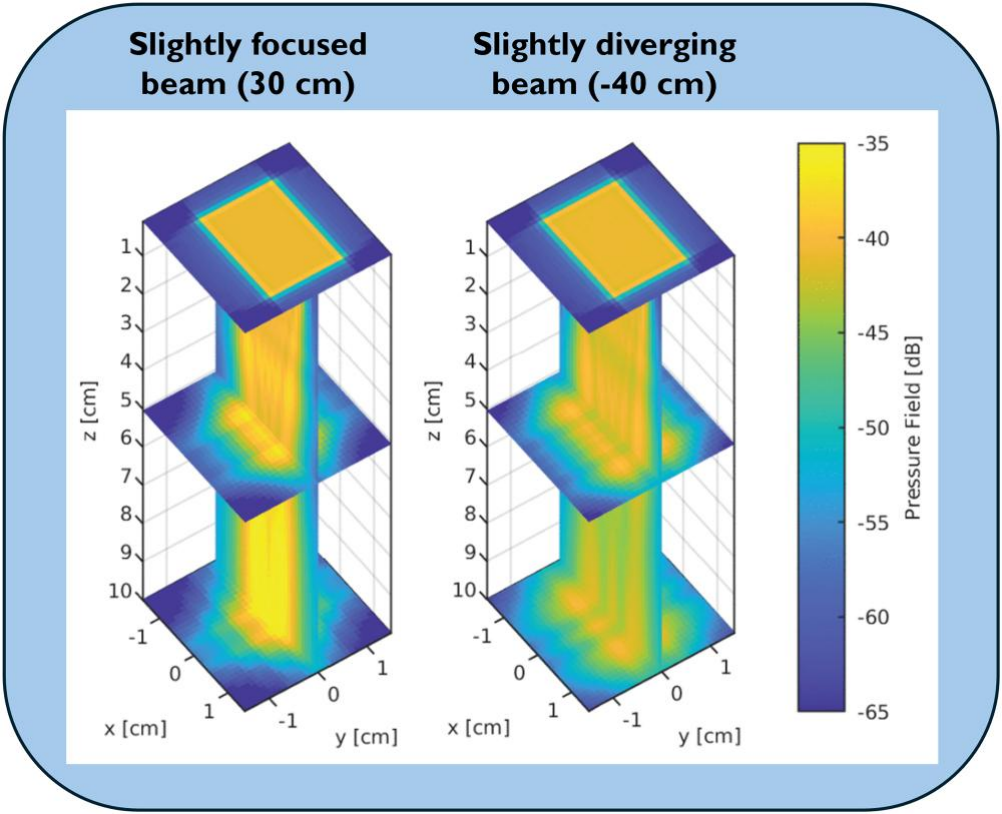
Effects on beam size and energy from different focusing settings at typical imaging depth. Computer simulations at transmit frequency 2.0 MHz. In this clinical validation, we applied a slightly focused beam (*Left*), producing a narrower beam with more focused energy and stronger scattered signal than a slightly diverging beam (*Right*). Adapted from: *Fiorentini S. et al Quantification of Flow Rates and Flow Volumes in Valve Regurgitation Using 3-D High Frame-Rate Ultrasound.* IEEE Open Journal of Ultrasonics, Ferroelectrics, and Frequency Control. *2023;**3**:29–40*.^17^

Three-dimensional HPRFD data were acquired by transmit and receive on all elements of the 4V-D transducer, producing a single broad beam with a focus depth at 30 cm (*Figure 2*). High-pulse repetition frequency (HPRF) was enabled to prevent aliasing at the cost of spatial ambiguity. Transmit frequency was set as high as possible within 1.5–2 MHz depending on the jet depth and maximum velocity. The sample volume had a length of 2 cm. Each recording consisted of 24 000 frames, corresponding to at least one cardiac cycle at the typical pulse repetition frequency (PRF) of 12–18 KHz. The data were stored as unprocessed (channel) data and were processed and analysed off-line.

Three-dimensional HPRFD was defined as non-feasible if RVol calculation was impossible due to insufficient PRF, low signal-to-noise ratio, or in the absence of detectable flow.

### Echocardiographic reference

The reference echocardiographic assessment was based on comprehensive TTE recordings by an experienced echocardiographer. Analyses were performed using Echopac v.203 (GEVU). Regurgitation severity grading was performed by a comprehensive integrative approach in line with the 2017 ASE recommendations,^1^ similar to the 2022 EACVI recommendations.^2^ Chamber quantification was performed in accordance with current recommendations.^18^ Image quality was scored subjectively as good, moderate, below moderate, or poor.

Two-dimensional PISA RVol estimation was performed in conjunction with the comprehensive echocardiographic assessment. In MR, specific attention was drawn to variation in systolic regurgitant flow rate and corresponding variation in PISA and continuous wave Doppler density. In holosystolic regurgitation with markedly varying regurgitant flow rate, an average PISA from at least three time points was applied. If unfeasible, a representative average of PISA was sought. In the presence of two clearly separated jets, PISA was performed on both jets and the RVols added together. Proximal isovelocity surface area was defined as non-feasible in the presence of marked wall impingement or funnel shape of the flow convergence, or in the absence of either a detectable flow convergence or a continuous wave Doppler envelope.

### CMR reference

The CMR reference included steady-state free precession recordings of a short-axis (SAX) stack of the complete left ventricle (LV) and phase-contrast recordings from the aorta, all recorded during breath hold. The LV SAX stack had an 8 mm slice thickness without gaps. Centred in the iso-centre of the magnetic field, phase-contrast slices were recorded perpendicular to the aortic long axis at three levels at the aortic root and one additional level at the pulmonary artery. Slice thickness was 5 mm. Phase-contrast velocity encoding was assigned individually. Phase-contrast recordings of a large stationary sodium polyacrylate-based gel phantom were performed immediately after each patient scan using identical recording settings.

Cardiac magnetic resonance analyses were performed in accordance with current recommendations.^19^ Left ventricular end-diastolic volume, stroke volume (SV), and ejection fraction were estimated from the LV SAX stack. Trabeculae and papillary muscles were included in the cavity. Aortic SV and RVol were calculated from the estimated through-plane flow from the phase-contrast slice closest to the sino-tubular junction. Mitral RVol was calculated indirectly from the subtraction of aortic SV from LV SV. Phase-contrast recordings non-perpendicular to flow, with unstable heart cycle length from premature beats, or with aliasing were discarded. In such cases, the second closest slice to the sino-tubular junction or the slice at the pulmonary artery level was selected, in that order. Manually corrected automatic segmentations were used for both LV volumetrics and flow quantification. To account for magnetic field inaccuracies, we applied a second-degree polynomial correction using the synthetic gel phantom recordings as the reference.^20^ If gel phantom recordings were mispositioned or missing, a similar correction was applied using stationary patient tissue as the reference. All CMR analyses were performed in Segment v3.2 (Medviso, Lund, Sweden).^21^

### Statistical analyses

Dichotomous data are presented as n/total (%) or %. The Shapiro–Wilk test was used for testing normality of the data, along with histograms and QQ-plots. Continuous variables are presented as mean (±SD) or median (25th percentile to 75th percentile) as appropriate. Spearman’s rank correlation test was applied to assess correlation between non-normally distributed continuous data or combinations of continuous and categorical data and reported as the Spearman correlation coefficient, *rho*. To investigate the association between categorical data, Pearson’s χ^2^ or Spearman’s rank correlation tests were used as appropriate. A two-way mixed intraclass correlation model for consistency was applied to investigate agreement between continuous data and reported with an intraclass correlation coefficient. Cohen’s Kappa statistics were applied to investigate agreement between ordinal parameters and reported with the Kappa coefficient, *K*. For all analyses, a two-sided *P* < 0.05 was considered statistically significant. Data were organized and analysed in SPSS 28.0 (IBM, New York, NY, USA).

## Results

We included 83 patients, of which seven were included in both the AR and MR patient subsets, meaning both subsets included data from 45 patients. All participants underwent standard TTE, 3D HPRFD, and CMR. Median time between start of TTE and start of CMR was 1.5 h. Analyses were performed 10–18 months after the final inclusion.

### Patient characteristics

Patient characteristics and basic imaging metrics are provided in detail in *Table 1*. All patients were in sinus rhythm.

**Table 1:** Patient characteristics and basic imaging metrics

		AR (*n* = 45^a^)	MR (*n* = 45^a^)
Patient characteristics	Age (years)	60 (44–70)	60 (±13)
	Male sex	27/45 (60%)	25/45 (55%)
	Body mass index (kg/m^2^)	24.9 (23.8–26.7)	24.7 (±2.7)
	Body surface area (m^2^)	1.92 (±0.18)	1.89 (±0.20)
	Sinus rhythm	45/45 (100%)	45/45 (100%)
	Systolic blood pressure	144 (129–158)	142 (±20)
	Diastolic blood pressure	76 (72–78)	81 (±9)
TTE	Image quality ≥ moderate	37/45 (82%)	34/45 (76%)
	LV internal diameter, end-diastole (mm)	54 (±7)	53 (46–58)
	LV biplane EF (%)	62 (±5)	62 (±6)
	LV GLS (%)	−20 (±2)	−22 (±3)
	Left atrial volume index (mL/m^2^)	38 (31–47)	49 (36–64)
CMR	LV EDV (mL)	222 (±78)	191 (149–228)
	LV SV (mL)	125 (96–154)	117 (99–139)
	LV EF (%)	59 (±6)	60 (56–68)
Time between start of exams (h)		1.75 (1.5–1.75)	1.5 (1.25–1.5)

^a^Seven patients featured in both groups.

Details on regurgitation characteristics are displayed in *Table 2*. Based on the TTE assessment, 42% of AR patients had mild regurgitation, 40% had moderate regurgitation, and 18% had severe regurgitation. Thirty-eight percent of ARs were eccentric. Similarly, 67% of MR patients had mild regurgitation, 24% had moderate regurgitation, and 9% had severe regurgitation. Eighty-two percent of MRs were primary regurgitations and 40% were eccentric.

**Table 2:** Core regurgitation characteristics, feasibility, and RVol estimation

		AR	MR
TTE	Eccentric jet	17/45 (38%)	18/45 (40%)
	Multiple jets	3/45 (7%)	9/45 (20%)
	Significant within-beat variation of orifice size	0/45 (0%)	37/45 (82%)
	Valvular/annular sclerosis, any degree	34/45 (76%)	5/45 (11%)
	Non-tricuspid aortic valve	16/45 (36%)	
	Primary MR		37/45 (82%)
	PISA feasibility	39/45 (87%)	33/45 (73%)
	PISA effective regurgitant orifice area (mm^2^)	13 (9–20)	13 (9–25)
	PISA RVol (mL)	35 (22–51)	17 (13–34)
	Mild regurgitation (overall)	19/45 (42%)	30/45 (67%)
	Moderate regurgitation (overall)	18/45 (40%)	11/45 (24%)
	Severe regurgitation (overall)	8/45 (18%)	4/45 (9%)
CMR	Complete data for regurgitation quantification	43/45 (96%)	24/45 (53%)
	Phase-contrast velocity encoding (cm/s)	200 (170–230)	170 (140–170)
	Phase-contrast phantom correction	36/43 (84%)	20/24 (83%)
	RVol (mL)	15 (8–52)	17 (6–35)
3D HPRFD	Feasibility of RVol estimation, overall	25 (56%)	20 (44%)
	Feasibility based on TTE regurgitation severity	*P* = 0.02^a^	*P* = 0.02^a^
	Mild	14/19 (74%)	17/30 (57%)
	Moderate	9/18 (50%)	2/11 (18%)
	Severe	2/8 (25%)	1/4 (25%)
	Feasibility based on jet direction	*P* = 0.006^b^	*P* = 0.01^b^
	Central	20/28 (71%)	16/27 (59%)
	Eccentric	5/17 (29%)	4/18 (22%)
	Feasibility based on TTE overall image quality	*P* = 0.06^a^	*P* = 0.045^a^
	≥moderate	23/37 (62%)	18/34 (53%)
	<moderate	2/8 (25%)	2/11 (18%)
	RVol (mL)	41 (27–55)	16 (11–26)
	Jet cross-sectional area (mm^2^)	29 (28–62)	32 (23–41)

^a^Spearman’s rank correlation

^b^Pearson’s Chi-square.

### Feasibility

Details on feasibility are provided in *Table 2*.

Feasibility of RVol estimation by 3D HPRFD was 56% in AR and 44% in MR. The main causes of non-feasibility were low signal-to-noise ratio and insufficient PRF. Additionally, regurgitation severity and jet eccentricity impacted the feasibility of 3D HPRFD, which was only 25% in severe AR and MR, and 29% in eccentric AR and 22% in eccentric MR. Notably, jet eccentricity and regurgitation severity were related in both MR and AR (*P* < 0.001 for both). Finally, less than moderate TTE image quality was associated with a lower 3D HPRFD feasibility, although only reaching statistically significant levels in MR (*P* = 0.045), not in AR (*P* = 0.06).

Due to human error, essential parts of the CMR data for regurgitation quantification were not exported from the scanner to long-term storage for 2/45 (4%) patients in the AR and 21/45 (47%) patients in the MR subsets. As the Covid-19 pandemic led to a delay in data analyses, this error was not discovered until one year after completion of inclusions. Hence, recovery of these data was no longer possible. Regurgitant volume estimation by CMR was feasible in all cases with complete CMR data.

Feasibility of RVol estimation by 2D PISA was 87% in AR and 73% in MR.

### Correlation and agreement

**Figure 3:**
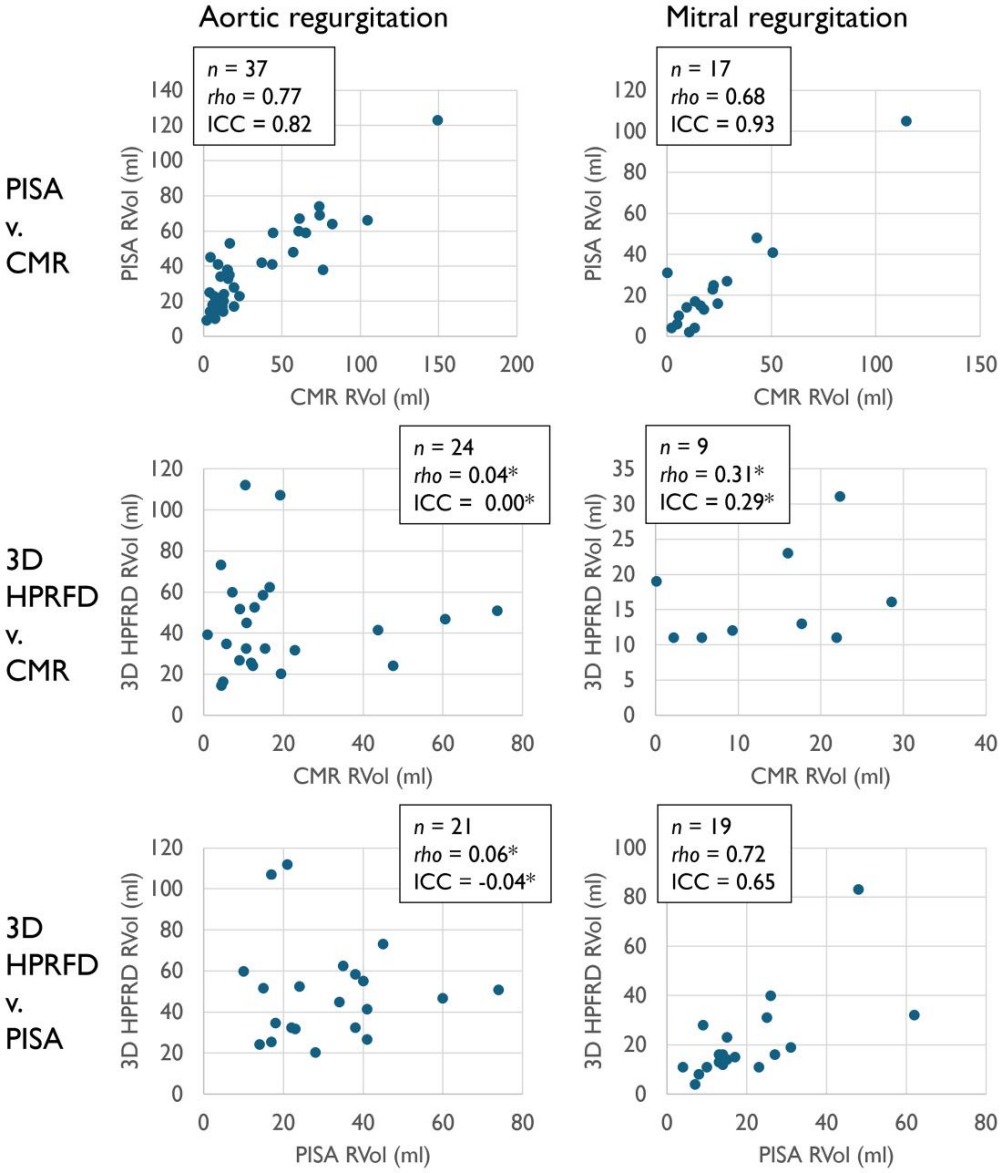
Scatter plots of RVol estimates from the 2D PISA method, CMR, and 3D HPRFD. Spearman rank correlation coefficient, rho, and ICC for consistency are reported for each plot. *n* equals the number of patients represented in each plot. *No statistically significant correlation (*P* ≥ 0.05).

*Figure 3* displays scatter plots of estimated RVol from 3D HPRFD, 2D PISA, and CMR.

In AR, RVol from 3D HPRF correlated neither with 2D PISA nor with CMR. Similarly, intraclass correlation demonstrated no statistically significant agreement.

In MR, RVol from 3D HPRFD correlated with 2D PISA (*rho* = 0.72). Moreover, intraclass correlation demonstrated a moderate agreement between RVol from 3D HPRFD and 2D PISA (ICC = 0.65). However, there was no significant rank correlation or intraclass correlation between RVol from 3D HPRFD and CMR, though, notably, only nine patients had both feasible 3D HPRFD and available CMR data.

**Figure 4:**
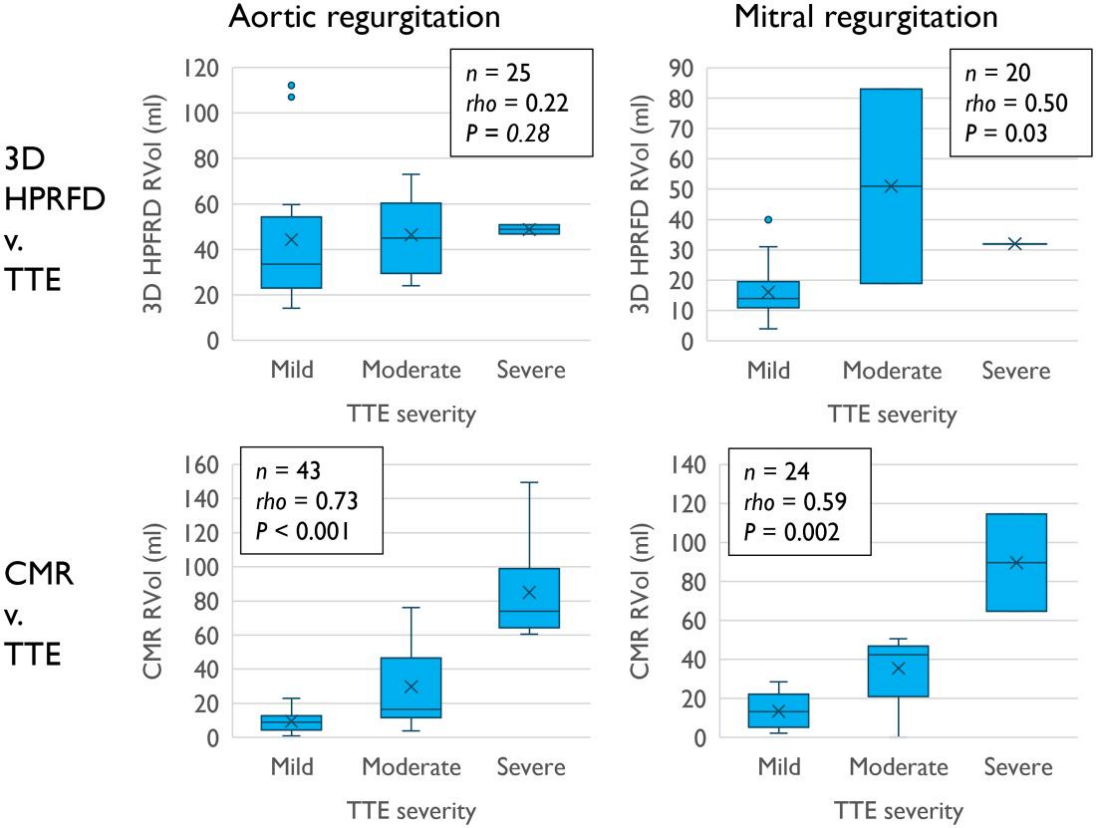
Box plots of RVol estimates from 3D HPRFD and CMR against overall severity grading by TTE. Spearman rank correlation coefficient, rho, and the corresponding *P* are reported for each plot. *n* equals the number of patients represented in each plot.

*Figure 4* displays box plots of estimated RVol from 3D HPRFD and CMR against overall regurgitation severity grading by TTE. In AR, there was no statistically significant rank correlation between RVol by 3D HPRFD and overall severity grading by TTE. In MR, these parameters correlated (*rho* = 0.50). However, in neither AR nor MR could 3D HPRFD RVol discriminate severe from non-severe regurgitation.

*Table 3* displays cross-tables between overall regurgitation severity grading by TTE and categorized RVol estimates by 3D HPRFD and CMR. Kappa statistics revealed no statistically significant agreement surplus to chance between categorized RVol estimates from 3D HPRFD and overall severity grading by TTE.

**Table 3:** Agreement between categorized RVol and TTE overall regurgitation severity

		CMR AR RVol (mL)						CMR MR RVol (mL)			
		<30	30–59	≥60	Total			<30	30–59	≥60	Total
TTE AR overall severity grade	Mild	19	0	0	19	TTE MR overall severity grade	Mild	17	0	0	17
	Moderate	9	5	2	16		Moderate	1	4	0	5
	Severe	0	0	8	8		Severe	0	0	2	2
	Total	28	5	10	43		Total	18	4	2	24
	*K*	0.59	*P*	<0.001			*K*	0.90	*P*	<0.001	

		3D HPRFD AR RVol (mL)						3D HPRFD MR RVol (mL)			
		<30	30–59	≥60	Total			<30	30–59	≥60	Total
TTE AR overall severity grade	Mild	5	6	3	14	TTE MR overall severity grade	Mild	15	2	0	17
	Moderate	2	5	2	9		Moderate	1	0	1	2
	Severe	0	2	0	2		Severe	0	1	0	1
	Total	7	13	5	25		Total	16	3	1	20
	*K*	0.06	*P*	0.65			*K*	0.17	*P*	0.33	

## Discussion

In this first clinical validation study of RVol estimation by 3D HPRFD in patients with AR and MR we found that the method had a low feasibility and poor rank correlation and agreement with estimated RVol by the 2D PISA and CMR references.

### 3D HPRFD—clinical utility

The overall feasibility of RVol estimation by 3D HPRFD was 50%, dropping to 25% in severe regurgitation. This is a major limitation of the method. In AR, we found no statistically significant rank correlation or agreement between 3D HPRFD and any of the references. In fact, the two largest estimated RVols by 3D HPRFD were seen in mild AR. In MR, the results were slightly more positive, including a statistically significant positive rank correlation and modest agreement between 3D HPRFD and 2D PISA RVol. However, due to the low feasibility of 3D HPRFD in more than mild MR, this included very few moderate and severe regurgitations, meaning these findings do not translate to clinical utility. Moreover, the cross tables and Kappa statistics (*Table 3*) and box plots (*Figure 4*) suggest that, in its current state, 3D HPRFD cannot discriminate severe from non-severe regurgitation.

### 3D HPRFD—technical considerations

The 3D HPRFD method was inspired by previous work on jet core assessment.^12–16^ In principle, it brings a one-shot acquisition without the need for power Doppler calibration. The method accounts for non-constant flow rate and non-circular orifice and is flow convergence independent. *In vitro*, consistent RVol estimates have been demonstrated for beam-to-jet angles from 0° to 50°.^17^ Moreover, artificial intelligence–based segmentation of the jet cross-sectional area has already been presented.^22^

For accurate RVol estimation, 3D HPRFD depends on complete capture of the jet core with a sufficiently high signal-to-noise ratio and maximum detectable velocity at the highest possible spatial resolution. These factors are inter-related, and there are important tradeoffs between: (i) beam width and scattered signal strength; (ii) PRF (maximum detectable velocity) and scattered signal strength; (iii) transmit frequency (maximum detectable velocity) and spatial resolution; and (iv) transmit frequency (maximum detectable velocity) and beam width.

*Figure 2* displays beam profiles with slightly focusing and slightly diverging beams. The focused beam, which was used in this study, returns a stronger scattered signal than the diverging beam. The drawback is a narrower beam with increased risk of incomplete capture of the jet core. Theoretically, very large jets may exceed the effective beam width, causing underestimation of RVol. Nevertheless, severe regurgitation produces a strong scattered signal which should enhance feasibility, which is opposite to what we found. This could be explained by the correlation between jet eccentricity and severity, e.g. in this study, 83% of severe regurgitations were eccentric. Although 3D HPRFD does not depend on perfect beam-to-jet alignment, beam-to-jet angle deviations >50° were avoided. In eccentric jets, beam-to-jet alignment causes valve plane excursion to displace the jet laterally relative to the beam direction, potentially causing incomplete jet core capture. A broader beam would be less vulnerable to this.

**Figure 5:**
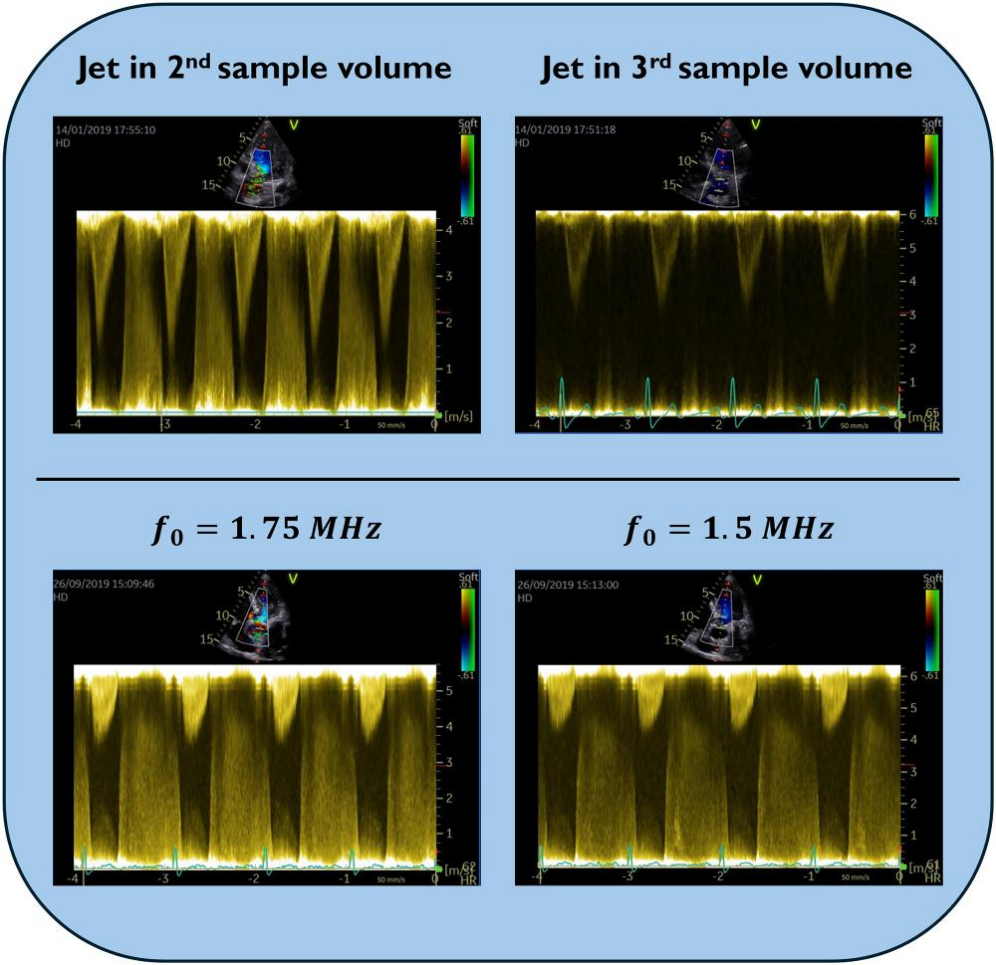
Increase in maximum detectable velocity. *Top left*: regurgitant jet maximum velocity cannot be discriminated from signals from low velocity flow or stationary tissue. Top right: increased PRF leads to an additional sample volume close to the transducer capturing strong reflectors of the apical region. This leads to signal saturation, resulting in signal loss from the sample volume containing the jet. Bottom: Reduced transmit frequency (f_0_) increases the maximum detectable velocity, allowing for better discrimination of the regurgitant jet maximum velocity envelope from signals from low velocity flow or stationary tissue. This comes at the costs of impaired spatial resolution and a narrower beam at the typical imaging depth.

High-pulse repetition frequency recordings are essential to estimating jet core velocities of left-sided regurgitations. Frequently, a sufficiently high PRF could not be obtained at the second sample volume at the default 2.0 MHz transmit frequency. Further PRF increase entailed an additional sample volume close to the transducer, covering strong reflectors in the LV apical area. This typically led to saturation,^23^ resulting in signal loss from the jet (*Figure 5*). Another way to increase the maximum detectable velocity is to reduce transmit frequency (*Figure 5*). However, this impairs spatial resolution. Moreover, from the effect of diffraction focusing, it also brings the effective focus point closer to the transducer, resulting in a narrower ultrasound beam at the typical imaging depth.

Applying a slightly diverging beam was successful *in vitro*, producing better results than a slightly focused beam.^17^ However, our pilot study in 10 patients with good image quality using the diverging beam settings resulted in a feasibility of only 40%. Hence, diverging the beam does not seem to provide a single solution to the *in vivo* challenges.

### Reference methods

In the presence of two clearly separated jets, PISA was performed on both and added together. In holosystolic regurgitation with markedly varying flow rate, an average PISA was applied. Although both approaches are theoretically logical, neither of them has been thoroughly validated.

In AR, 76% of the patients had some degree of aortic valve calcification. Assigned from systolic flow, CMR median velocity encoding was rather high at 200 (170–230) cm/s. This may impact the accuracy of AR RVol estimation by CMR.

### Statistical methods

None of the methods yielded normally distributed RVol estimates, hence, we report rank correlation. For the applied intraclass correlation model, some deviation from normal distribution is acceptable.^24^

### Future perspectives

Three-dimensional HPRFD post-processing could be developed to handle multiple jets and to function fully automatically.

However, future work should focus on optimizing the acquisition sequence. Ideally, the acquisition should provide a broader beam without compromising the signal-to-noise ratio. Moreover, a sufficiently high maximum detectable velocity should be achieved without compromising lateral resolution, and saturation from the strong reflectors in the LV apical region should be avoided. Future increases in transducer dynamic range and improved scanning efficiency may help solve these challenges.

Three-dimensional HPRFD application in right-sided regurgitations, shunts, or TOE remains unexplored.

### Study limitations

This study is quite small. Cardiac magnetic resonance reference was available in 96% of AR patients but only 53% of MR patients. Despite our efforts to balance the distribution of TTE severity grade, this was not fully achieved. This was partly due to pre-inclusion overestimation of regurgitation severity from standard clinical care. Moreover, severe regurgitation is often associated with arrhythmia, which was an exclusion criterion. Similarly, patients unable to lie flat were excluded. To limit patient factors that may cause inaccurate flow quantification by CMR, patients with metal implants such as cardiac devices, prosthetic valves or sternal cerclage were excluded. The study exclusion criteria may also explain the four-to-one imbalance between primary and secondary MR. Moreover, the low feasibility of 3D HPRFD, especially in severe regurgitation, effectively lowered the sample size further. Consequently, only two patients with severe AR and one patient with severe MR had a feasible 3D HPRFD RVol estimate, which is an important limitation of the study.

## Conclusions

A 3D HPRFD method for quantification of RVol from TTE was highly successful *in vitro*. However, results from the present clinical validation study in patients with AR and MR were disappointing, demonstrating a low feasibility for RVol estimation, particularly in severe regurgitation. Overall, 3D HPRFD RVol estimates correlated poorly with TTE and CMR references, and the method could not discriminate severe from non-severe regurgitation. Despite the limitations of this small study, the results clearly demonstrate that 3D HPRFD is currently not ready for clinical use.

Significant improvements in 3D HPRFD data quality must be obtained before the method may provide a clinically useful tool for quantification of left-sided valvular regurgitation. However, the combination of high jet velocities, imaging depth, and strong ultrasound reflectors in the apical region constitutes a challenge that may not be easily solved on current commercial ultrasound systems.

## Data Availability

Data may be shared upon reasonable request to the corresponding author.

## Lead author biography

Erik Andreas Rye Berg (b. 1981), MD, graduated from Norwegian University of Science and Technology (NTNU) in 2008. He has performed research at Centre for Innovative Ultrasound Solutions and NTNU since 2016. Since 2017, he has been a consultant in internal medicine at St. Olavs hospital, Orkdal, Norway. He currently holds a position as fellow in cardiology at St. Olavs hospital, Trondheim, Norway. His main scientific interests are related to myocardial function and valvular heart disease.
